# Associations Between Soluble Cell Adhesion Molecules and Cardiovascular Comorbidities in Systemic Sclerosis: Implications for Insulin Resistance

**DOI:** 10.3390/jcm14051467

**Published:** 2025-02-22

**Authors:** Iván Ferraz-Amaro, Zeina Ibrahim-Achi, Antonia de Vera-González, Alejandra González-Delgado, Mónica Renuncio-García, Esther F. Vicente-Rabaneda, J. Gonzalo Ocejo-Vinyals, Santos Castañeda, Miguel Á. González-Gay

**Affiliations:** 1 Division of Rheumatology, Hospital Universitario de Canarias, 38320 Tenerife, Spain; 2 Department of Internal Medicine, University of La Laguna (ULL), 38200 Tenerife, Spain; 3 Division of Angiology and Vascular Surgery, Hospital Universitario de Canarias, 38320 Tenerife, Spain; zenaibrahimachi@gmail.com; 4 Division of Central Laboratory, Hospital Universitario de Canarias, 38200 Tenerife, Spain; adeverag@gmail.com (A.d.V.-G.); alejandra.gd88@gmail.com (A.G.-D.); 5 Division of Immunology, Hospital de León, 24008 León, Spain; mrenuncio@saludcastillayleon.es; 6 Division of Rheumatology, Hospital Universitario de La Princesa, IIS-Princesa, 28006 Madrid, Spain; efvicenter@gmail.com (E.F.V.-R.); scastas@gmail.com (S.C.); 7 Division of Immunology, Hospital Universitario Marqués de Valdecilla, Instituto de Investigación Valdecilla (IDIVAL), 39008 Santander, Spain; javiergonzalo.ocejo@scsalud.es; 8 Division of Rheumatology, IIS-Fundación Jiménez Díaz, 28006 Madrid, Spain; 9 Medicine and Psychiatry Department, University of Cantabria, 39005 Santander, Spain

**Keywords:** systemic sclerosis, soluble cell adhesion molecules, insulin resistance, cardiovascular disease

## Abstract

**Background**: Soluble cell adhesion molecules such as sICAM-1 (soluble intercellular adhesion molecule-1), sVCAM-1 (soluble vascular cell adhesion molecule-1), and P-selectin have been implicated in cardiovascular disease pathogenesis in the general population. Cardiovascular disease is prevalent among patients with systemic sclerosis (SSc). This study aims to investigate potential associations between the serum levels of these adhesion molecules and specific cardiovascular comorbidities in SSc patients. **Methods**: This cross-sectional study encompassed 81 individuals with SSc. All SSc patients underwent a complete clinical evaluation. Serum sICAM-1, sVCAM-1, and P-selectin levels, lipid profiles and insulin resistance indices, and carotid ultrasound were assessed. Multivariable linear regression analyses were employed to investigate potential associations between adhesion molecule levels (sICAM, sVCAM, and P-selectin) and both SSc-specific manifestations and cardiometabolic parameters. **Results**: The associations of disease-related parameters with sICAM-1, sVCAM-1, and P-selectin levels were limited. Notably, only the modified Rodnan skin score exhibited a significant positive association with sVCAM-1 levels, while no such associations were observed for sICAM-1 and P-selectin. Regarding cardiovascular disease-related data, sVCAM-1 significantly correlated with higher values of insulin resistance and beta-cell function indices. In the case of P-selectin, although a trend was observed, statistical significance was not reached. **Conclusions**: In patients with SSc, serum values of sVCAM-1 independently correlate with insulin resistance. The assessment of CAMs in patients with SSc could serve as a valuable clinical tool for identifying individuals with increased insulin resistance and a higher risk of cardiovascular disease.

## 1. Introduction

Systemic sclerosis (SSc), commonly known as scleroderma, is a complex chronic autoimmune disorder characterized by widespread vascular abnormalities and progressive fibrosis. This multisystem disease affects not only the skin, but also various internal organs. The hallmark clinical manifestations of SSc include extensive skin thickening and hardening, accompanied by Raynaud’s phenomenon, all of which are observed in virtually all patients diagnosed with this condition. However, this condition is notably heterogeneous, as reflected in its diverse range of organ manifestations variable disease progression, severity, and clinical outcomes [1,2]. SSc is generally classified according to the extent of skin involvement, patterns of internal organ involvement, and the presence of overlapping features with other systemic rheumatic disease systems. Based on these criteria, the primary subsets of SSc include limited cutaneous, diffuse cutaneous, SSc sine scleroderma, and SSc overlap syndrome [3].

Cardiovascular disease is common but often underrecognized in patients with SSc [4]. Contributing factors to vascular issues in SSc patients which overlap with those seen in relation to atherosclerosis include endothelial dysfunction, a reduced number of circulating endothelial progenitor cells, and an increase in microparticles [4]. A systematic review and meta-analysis compared atherosclerosis risk between SSc patients and healthy individuals, revealing that SSc patients show significantly higher carotid intima-media thickness (cIMT) and reduced flow-mediated vasodilation [5]. Similarly, metabolic syndrome is relatively common in patients with SSc, potentially exacerbating the risk of cardiovascular disease [6]. In addition, SSc patients exhibit abnormal lipid profiles compared to healthy controls, including reduced cholesterol efflux capacity [7]. Furthermore, insulin resistance has been independently linked to the presence of digital ulcers in SSc patients and may serve as a potential biomarker for microvasculopathy in these patients [8].

Soluble cell adhesion molecules (sCAMs) are a class of cell adhesion molecules that play crucial roles in various physiological and pathological processes. Although direct measurement of cellular adhesion molecules (CAMs) expression presents significant clinical challenges, their soluble counterparts can be readily detected in the bloodstream. These soluble forms can be quantified in serum or plasma, potentially serving as surrogate markers for CAM expression and activity. Soluble isoforms of the CAMs are mainly sICAM-1 (soluble intercellular adhesion molecule-1), sVCAM-1 (soluble vascular cell adhesion molecule-1), and P-selectin. Although belonging to the same adhesion molecule family, ICAM-1, VCAM-1, and P-selectin exhibit distinct characteristics. ICAM-1 is expressed on multiple cell types, facilitating leukocyte recruitment across various subtypes [9]. VCAM-1, primarily found on activated endothelial cells, specializes in lymphocyte and monocyte adhesion [10]. P-selectin, stored in Weibel–Palade bodies, enables the initial tethering and rolling of neutrophils and monocytes during inflammatory processes [11].

CAMs have emerged as important biomarkers in cardiovascular disease. For example, sVCAM-1 and sICAM-1 have been shown to be significantly related to future death from cardiovascular causes among patients with documented coronary artery disease [12]. Similarly, elevated levels of sICAM-1 have been associated with increased subclinical atherosclerosis and progression of coronary artery calcification and carotid artery stenosis [13], with higher sICAM-1 correlating with an increased risk of all-cause mortality and cardiovascular disease-specific mortality in older adults [14]. Likewise, sVCAM-1 has been proposed as a biomarker to predict the occurrence, development, and preservation of cardiovascular disease [15]. Lastly, P-selectin plays a key role in the pathogenesis of cardiovascular diseases through their involvement in leukocyte adhesion and inflammation [16,17].

This study aims to investigate the potential associations between the serum levels of sICAM-1, sVCAM-1, and P-selectin and specific cardiovascular comorbidities in patients with SSc. The research focuses on examining relationships with metabolic syndrome, lipid profiles, insulin resistance, and subclinical carotid atherosclerosis. Our study contributes to the understanding of mechanisms associated with cardiovascular disease in patients with SSc.

## 2. Materials and Methods

### 2.1. Study Participants

This cross-sectional investigation encompassed 81 individuals diagnosed with SSc, all of whom were adults aged 18 years or above. Each participant fulfilled the 2013 classification criteria for SSc as jointly established by the American College of Rheumatology and the European League Against Rheumatism. These criteria served as the definitive standard for patient inclusion in our study cohort [18]. All participants had received their SSc diagnosis from qualified rheumatologists and were under regular follow-up care at our institution’s rheumatology outpatient clinics. To be eligible for inclusion in this study, patients were required to have a disease duration of at least one year. We excluded individuals with a history of cardiovascular events, cancer, or any other chronic conditions. Additionally, patients showing signs of active infection or those with a glomerular filtration rate below 60 mL/min/1.73 m^2^ were not included in the study cohort. We believe that the presence of these other disease could have introduced a bias because they could modify CAMs serum levels. The study protocol was approved by the Institutional Review Committee at the Hospital Universitario de Canarias and all subjects provided informed written consent (approval number: CHUC_2019_49).

### 2.2. Disease-Related Data Collection

Localized and diffuse forms of SSc were determined based on the extent of skin thickening. The localized form was diagnosed if skin sclerosis was present distal to the elbows and knees and, to a lesser extent, on the face and neck, while the trunk and proximal extremities were spared. Surveys in SSc patients were performed to assess cardiovascular risk factors and medication use. Subjects completed a questionnaire and underwent a physical examination to determine anthropometric measurements and blood pressure. Medical records were reviewed to ascertain specific diagnoses, medications, and comorbidities. Hypertension was defined as a blood pressure consistently at or above 140/90 mmHg. Obesity was classified as a body mass index (BMI) of 30 kg/m^2^ or higher. For the purposes of this study, SSc disease duration was calculated from the onset of the first non-Raynaud’s SSc-related symptom. The modified Rodnan skin score (mRSS) was employed to quantify the degree of skin thickening in SSc patients. This validated assessment tool evaluates skin thickness at multiple body sites, providing a comprehensive measure of cutaneous involvement in SSc [19]. This score has been widely adopted as a primary outcome measure in clinical trials for SSc. This validated assessment tool evaluates skin thickness in 17 distinct anatomical areas, with each site scored from 0 (normal) to 3 (severe), demonstrating acceptable intra-rater reliability. Esophageal involvement in SSc was defined by the presence of dysmotility on manometric evaluation. Articular manifestations were identified through clinical examination, including evidence of joint swelling, deformities, contractures, and tendon friction rubs. Interstitial lung disease (ILD) was diagnosed based on pulmonary function tests and imaging criteria. Specifically, ILD was defined by a predicted forced vital capacity (FVC) of ≤80%, a forced expiratory volume in one second to FVC ratio (FEV1/FVC) of ≥70%, and/or a predicted diffusing capacity for carbon monoxide (DLCO) of <80% in conjunction with interstitial changes observed on high-resolution computed tomography (HRCT) of the chest. Nailfold capillaroscopy was performed as previously described [20] and scleroderma patterns were subgraded as “early”, “active”, and “late” [20].

### 2.3. Subclinical Carotid Atherosclerosis Assessment and SCORE2 Calculation

The cardiovascular risk score (SCORE2) was calculated following the 2021 European Society of Cardiology guidelines for cardiovascular disease prevention in clinical practice [21]. SCORE2 classifies risk into low to moderate, high, or very high categories, with stratification based on age groups (<50, 50–69, and ≥70 years). This scoring system is specifically designed to estimate the 10-year risk of both fatal and non-fatal cardiovascular events in individuals aged 40 to 69 years. For healthy individuals aged 70 years or older, the SCORE2-OP (older persons) algorithm is used, providing estimates for both the 5-year and the 10-year risk of fatal and non-fatal cardiovascular events.

A carotid ultrasound examination was conducted to assess the carotid intima-media thickness (cIMT) within the common carotid artery. The aim was to detect the presence of localized plaques in the extracranial carotid arteries, which are located outside the skull. The measurements were performed using the EsaoteMylab 70 ultrasound system, manufactured in Genova, Italy. This system is equipped with a 7–12 MHz linear transducer and utilizes the quality intima-media thickness in real-time (QIMT) automated software-guided radiofrequency technique, developed by Esaote in Maastricht, Holland. The evaluation process followed the guidelines outlined in the Mannheim consensus [22], which defines the criteria for identifying plaques in the accessible extracranial carotid arteries. These arteries include the common carotid artery, the bulb, and the internal carotid artery. Plaque identification was based on the presence of a focal protrusion into the arterial lumen, with a carotid intima-media thickness (cIMT) measurement exceeding >1.5 mm. Additionally, the protrusion had to be at least 50% larger than the surrounding cIMT or cause a reduction of the arterial lumen of >0.5 mm [22].

### 2.4. Laboratory Assessments

Fasting serum samples were collected and frozen at −80 °C until analysis. Cholesterol, triglycerides, and high-density lipoprotein (HDL) cholesterol were measured using the enzymatic colorimetric assay (Roche). Low-density lipoprotein (LDL) cholesterol was calculated using the Friedewald formula. Dyslipidemia was recorded as occurring if one of the following was present: total cholesterol > 200 mg/dL, triglycerides > 150 mg/dL, HDL-cholesterol < 40 in men or <50 mg/dL in women, or LDL-cholesterol > 130 mg/dL. A standard technique was used to measure high-sensitivity C-reactive protein (CRP). Insulin resistance (IR) was assessed using the homeostatic model assessment (HOMA) method. Specifically, the HOMA model allowed for the estimation of insulin sensitivity (%S) and β-cell function (%B) based on fasting plasma insulin, C-peptide, and glucose concentrations. In this study, the updated HOMA2 computer model was utilized [23]. This model evaluates insulin sensitivity and β-cell function using paired fasting plasma glucose and specific insulin or C-peptide concentrations, covering ranges of 1–2200 pmol/L for insulin and 1–25 mmol/L for glucose. C-peptide is a more accurate marker for estimating β-cell function due to its role as a secretion indicator, while insulin data are preferred for calculating %S, as HOMA-%S is derived from glucose disposal relative to insulin concentration. In our study, IR and %S were calculated using serum insulin levels, whereas %B was determined using serum C-peptide levels. The HOMA2 computer model provided insulin sensitivity values expressed as HOMA2-%S (where 100% represents normal sensitivity). HOMA2-IR (insulin resistance index) was calculated as the reciprocal of %S. Insulin (Architect Abbott, 2000I, Abbott Park, Illinois, IL, USA) and C-peptides (Immulite 2000, Siemens, Tarrytown, NY, USA) were determined by chemiluminescent immunometric assays. sICAM-1, sVCAM-1, and P-selectin serum levels were measured by the electrochemiluminescence immunoassay method (MERCK^®^ MILLIPLEX map Multiplex Detection). Both the intra- and inter-coefficients of variability were <10% for these assays.

### 2.5. Statistical Analysis

Demographic and clinical characteristics of SSc patients and controls are presented as the mean (standard deviation) or as percentages for categorical variables. For continuous variables that did not follow a normal distribution, data were reported as median and first and third quartile ranges (Q1–Q3). The normality of the data was assessed using histogram plots and the Shapiro–Wilk test. The association between disease-related data and sICAM-1, sVCAM-1, and P-selectin was examined using multivariable linear regression analysis, with adjustments made for confounding variables. Confounders were selected from demographics if their *p*-values were below 0.20 in the univariable analysis of sICAM, sVCAM, and P-selectin. A *p*-value threshold of 0.20 was used to allow for the inclusion of variables that may have a meaningful impact on the outcome. This approach decreases the likelihood of erroneously excluding important confounders. Consequently, this threshold strikes a balance between including too many variables (which can lead to over-fitting) and too few (which can result in residual confounding). All analyses were conducted using the Stata software, version 17/SE (StataCorp, College Station, TX, USA), with a two-sided significance level set to 5%. A *p*-value less than 0.05 was considered statistically significant.

## 3. Results

### 3.1. Demographic, Laboratory, and Disease-Related Data in Patients with SSc

The characteristics of the SSc population are described in Table 1. The mean age was 60 ± 11 years, with 94% of the patients being women. Cardiovascular risk factors were prevalent in the patient sample: 40% had hypertension, 9% were smokers, and 9% had diabetes. Additionally, 25% took statins and 25% took aspirin.

Regarding the specific characteristics of the disease, 81% of the SSc patients had the limited type and 19% had the diffuse type. The mean age at recruitment was 60 ± 10 years and the duration of the disease was 8 (4–11) years. The median mRSS score was 4 (1–8). The presence of digital ulcers and calcinosis was reported in 15% and 19% of the patients, respectively. At the time of the study, 16% of the patients were taking prednisone, with a median dose of 5 (5–7.5) mg/day, and 5% were taking methotrexate. Additionally, 55 patients (72%) tested positive for anticentromere antibodies, and 11 patients (14%) tested positive for anti-Scl70 antibodies. Other disease-related characteristics are shown in Table 1.

### 3.2. Demographics and Disease Related Data Association with sICAM-1, sVCAM-1, and P-Selectin

The relationships among demographic characteristics, cardiovascular risk factors, and disease-related data with sICAM-1, sVCAM-1, and P-selectin values are shown in Table 2. With respect to this, while sex showed no association with these soluble adhesion molecules or P-selectin levels, age demonstrated a significant positive correlation with sVCAM-1. Body mass index, as well as waist and hip circumferences, showed a significant positive relationship with sICAM-1 levels. In contrast, the presence of traditional cardiovascular risk factors and the use of statins or aspirin did not correlate with sICAM-1, sVCAM-1, or P-selectin values (Table 2).

With respect to the clinical and laboratory characteristics of the disease, as well as the use of various therapies, no associations with sICAM-1, sVCAM-1, or P-selectin were observed. Specifically, the presence of arthritis, digital ulcers, pulmonary disease, and esophageal involvement were not related to CAMs. Only the Rodnan skin score demonstrated a significant positive association with sVCAM-1 values. Additionally, the use of NSAIDs and methotrexate showed significant positive relationships with P-selectin and sICAM-1, respectively (Table 2).

### 3.3. Association of Cardiometabolic Features with sICAM-1, sVCAM-1, and P-Selectin

Sixty-one of the patients met the criteria for metabolic syndrome and 34% had carotid plaques on carotid ultrasound. Additionally, the median SCORE2 was 4 (2–7) (Table 3). The lipid profile and insulin resistance indices are also shown in Table 3.

The presence of metabolic syndrome, carotid atheromatosis in the form of plaque or IMT, the SCORE2 cardiovascular risk calculator, and the lipid profile, together with insulin resistance indices, did not show significant relationships with sICAM-1 values. However, sVCAM-1, although not showing significant and consistent relationships with lipid profiles, showed several positive associations with insulin resistance indices. Specifically, serum sVCAM-1 levels demonstrated a positive relationship with serum insulin levels, C-peptide, and insulin resistance indices HOMA2-IR and HOMA2-B% after multivariable adjustment. This was also the case when exclusively nondiabetic patients were considered (Table 3). For P-selectin, the results regarding insulin resistance indices were similar, but, in this case, although a trend was observed, statistical significance was not reached (Table 3).

## 4. Discussion

Our study is the first in the literature to investigate the relationship between soluble CAMs and cardiovascular disease-related characteristics in patients with SSc. Based on our results, sVCAM-1 is associated with insulin resistance in SSc patients.

Insulin resistance has been reported to be more prevalent in SSc patients compared to healthy controls. A case–control study involving 73 SSc patients and 109 sex- and age-matched healthy controls has demonstrated significantly higher HOMA-IR values in SSc patients [8]. Notably, this study reveals an independent association between insulin resistance and the presence of digital ulcers in SSc patients. This finding underscores the potential relationship between insulin resistance and microvascular complications in SSc patients. The levels of insulin resistance (HOMA2-IR = 1.3) and beta-cell function (HOMA2-B% = 181) in our study sample corroborate previous reports of increased insulin resistance in SSc patients [8,24].

In a recent meta-analysis, patients with SSc have been described to have higher levels of the circulating cell adhesion molecules ICAM-1, VCAM-1, PECAM-1, E-selectin, and P-selectin compared to healthy controls [25]. These findings suggest that endothelial activation may play a central role in the pathogenesis of SSc. In this regard, positive immunostaining for E-selectin and ICAM-1 has been demonstrated on the endothelial cells of SSc patients’ skin but not in the controls’ skin [26]. Similarly, elevated levels of soluble VCAM-1, E-selectin, and ICAM-1 have been found in patients with SSc renal crisis, but not in those with SSc-associated pulmonary disease [27]. Also, one study of serial serum samples has indicated a correlation between changes in sVCAM-1 and in E-selectin and clinical deterioration or improvement in SSc patients [28]. However, these reports have generally come from small cross-sectional studies of patients with SSc focused on investigating a specific clinical manifestation. In our study, the patient inclusion criteria resulted in 81 subjects, and we evaluated multiple disease characteristics related to a wide range of organ manifestations. This comprehensive approach allows for a broader perspective on the relationships between CAMs and diverse aspects of SSc. In this sense, in our study, the association between sICAM-1, sVCAM-1, and P-selectin and specific clinical manifestations of systemic sclerosis was scarce. Only sVCAM-1 demonstrated a significant positive association with the mRSS, a measure of skin thickness.

Regarding cardiovascular disease aspects related to SSc, we did not find an association between any of the CAMs and the presence of metabolic syndrome, subclinical carotid atheromatosis, SCORE2 index, or lipid profile. However, notably, a positive relationship was observed between sVCAM-1 and P-selectin and insulin resistance indices. Although the association with P-selectin did not reach statistical significance, a trend was observed. The relationship between sVCAM-1 and insulin resistance indices aligns with previous studies in other populations, indicating that this association might be a common feature across various metabolic and inflammatory conditions including SSc. In this regard, insulin resistance and metabolic syndrome have been described to be associated with sICAM-1 in the general population [29] and in patients with systemic lupus erythematosus [30]. This has also been the case for sVCAM-1, which has been found to be lower in insulin-sensitive compared to insulin-resistant patients [31]. Similarly, research indicates a significant relationship between insulin resistance and selectins. For example, P-selectin deficiency has been found to protect against high-fat diet-induced obesity and insulin resistance [32], while, in healthy individuals, insulin resistance correlates positively with soluble E-selectin levels, independent of other factors like age and BMI [33].

In our study, the relationship between CAMs and insulin resistance was primarily observed with sVCAM-1 and P-selectin, but not with sICAM-1. We lack a definitive explanation for this observation. This differential association may be due to different levels of expression or shedding of these CAMs in SSc patients. For example, sVCAM-1 and P-selectin might be more prominently upregulated or cleaved in response to vascular inflammation characteristic of SSc, whereas sICAM-1 might remain relatively unaffected. On the other hand, it is possible that sVCAM-1 and P-selectin may be part of signaling pathways that are more directly linked to metabolic dysregulation and insulin resistance. In contrast, sICAM-1 might be primarily involved in other pathways less related to glucose metabolism, such as immune cell adhesion and transmigration. Furthermore, differences in post-translational modifications, such as glycosylation, may affect the stability, activity, or detection of CAMs. In this regard, sVCAM-1 and P-selectin could exhibit modifications in SSc patients that enhance their association with insulin resistance, unlike sICAM-1.

The strengths of our study include the comprehensive assessment of three CAMs, the robust evaluation of specific patients’ characteristics, and the comprehensive assessment of a full lipid profile, subclinical atherosclerosis, and insulin resistance indices including beta-cell function through C-peptide analysis. The limitations include the cross-sectional design that prevents the inference of causality. We selected this cross-sectional design as it serves to generate hypotheses that can subsequently be tested and confirmed in prospective studies. Therefore, future prospective studies should be conducted to confirm and extend our observations. Furthermore, our study focused on three CAMs known to be involved in major integrin pathways. However, there exists a wide array of other CAMs that were not evaluated in the present investigation. In addition, only one physician performed the mRSS assessments. This approach may have introduced potential bias in the determination of this score. Furthermore, as our study predominantly included female subjects, reflecting the fundamental female predominance of SSc, our results cannot be entirely generalized to male patients.

We did not include controls in our study. This could be considered a limitation. Nevertheless, we were not focused on comparing CAMs values between patients and controls, but rather on analyzing the relation that these have with disease characteristics in SSc subjects.

## 5. Conclusions

Our findings suggest a potential link between endothelial dysfunction markers and insulin resistance in SSc patients. We hypothesize that, eventually, this may contribute to the increased cardiovascular risk described in SSc. The measurement of CAMs in patients with SSc could potentially become a valuable clinical tool for identifying patients with elevated insulin resistance and at high risk of cardiovascular disease. Further research could explore whether targeting CAMs or related pathways could improve insulin sensitivity or reduce cardiovascular risk in SSc patients.

## Figures and Tables

**Table 1:** Demographics of systemic sclerosis patients.

		Scleroderma
		(n = 81)
Demographics		
Female, n (%)		76 (94)
Age, years		60 ± 11
BMI, kg/m^2^		29 ± 6
Waist circumference, cm		98 ± 14
Hip circumference, cm		106 ± 11
Waist to hip ratio		0.92 ± 0.09
Cardiovascular comorbidity		
Hypertension, n (%)		32 (40)
Current smoking, n (%)		7 (9)
Diabetes, n (%)		7 (9)
BMI > 30 kg/m^2^, n (%)		26 (32)
Statins, n (%)		20 (25)
Aspirin, n (%)		22 (27)
Systemic-sclerosis-related data		
SSc type, n (%)		
	Limited, n (%)	66 (81)
	Diffuse, n (%)	15 (19)
Disease duration, years		8 (4–11)
Modified Rodnan skin score, units		4 (1–8)
Raynaud phenomenon, n (%)		72 (90)
Calcinosis, n (%)		13 (16)
Digital ulcers, n (%)		12 (15)
Arthritis, n (%)		8 (10)
Gastric reflux, n (%)		41 (51)
Pathological esophageal manometry, n (%)		18 (55)
Nailfold capillaroscopy pattern		
	Normal	16 (22)
	Early	24 (33)
	Active	11 (15)
	Late	2 (3)
	Unclassified or not valuable	19 (26)
Interstitial lung disease, n (%)		13 (17)
	DLCO, %	75 ± 20
	FVC, %	93 ± 18
	FEV1, %	100 ± 18
Pulmonary hypertension, n (%)		12 (18)
Anti-centromere antibody positivity, n (%)		55 (72)
Anti-Scl70 antibody, n (%)		11 (14)
Therapies		
	Current NSAIDs, n (%)	8 (11)
	Current prednisone, n (%)	13 (16)
	Prednisone, mg/day	5 (5–7.5)
	Methotrexate, n (%)	4 (5)
	Chloroquine, n (%)	4 (5)
	Bosentan, n (%)	3 (4)

Data represent the mean ± SD or the median (Q1–Q3) when the data are not normally distributed. The esophageal manometry assessment was available only for 33 patients. BMI: body mass index; SSc: systemic sclerosis; NSAIDs: non-steroidal anti-inflammatory drugs. FVC: forced vital capacity; FEV: forced expiratory volume; DLCO: diffusion capacity of the lung for the carbon monoxide.

**Table 2:** Demographics and disease-related data association with sICAM-1, sVCAM-1, and P-selectin.

		sICAM-1	sVCAM-1	P-Selectin
		119 ± 56 ng/mL	755 ± 270 ng/mL	71 ± 27 ng/mL
		Beta Coefficient (95% Confidence Interval), *p*		
Demographics				
Female		−45 (−95–6), 0.082	−106 (−355–143), 0.40	−21 (−46–4), 0.10
Age, years		0.9 (−0.3–2), 0.14	**7 (2–13), 0.008**	−0.3 (−0.9–0.3), 0.34
BMI, kg/m^2^		**3 (1–5), 0.001**	9 (−0.9–20), 0.076	0.4 (−0.7–1), 0.49
Waist circumference, cm		**1 (0.4–2), 0.006**	3 (−1–8), 0.18	0.2 (−0.3–0.6), 0.50
Hip circumference, cm		**2 (0.5–3), 0.007**	3 (−2–9), 0.25	0.02 (−0.6–0.6), 0.95
Waist to hip ratio		88 (−49–224), 0.20	263 (−426–952), 0.45	37 (−32–106), 0.29
Cardiovascular comorbidity				
Hypertension		−13 (−39–13), 0.32	31 (−95–158), 0.62	4 (−9–16), 0.57
Current smoking		−8 (−52–36), 0.71	−0.7 (−215–214), 0.99	15 (−6–37), 0.16
Diabetes		−23 (−67–21), 0.30	−138 (−350–75), 0.20	−0.4 (−22–21), 0.97
BMI > 30 kg/m^2^		18 (−9–44), 0.20	2 (−130–134), 0.98	5 (−9–18), 0.50
Statins		12 (−17–41), 0.43	−49 (−190–91), 0.49	0.5 (−14–15), 0.94
Aspirin		−15 (−44–15), 0.32	63 (−80–205), 0.38	0.7 (−14–15), 0.92
Systemic-sclerosis-related data				
SSc type				
	Limited	ref.	ref.	ref.
	Diffuse	−16 (−48–16), 0.32	−31 (−187–125), 0.69	−4 (−20–12), 0.61
Disease duration, years		0.1 (−2–2), 0.91	7 (−4–18), 0.20	0.1 (−0.9–1), 0.82
Modified Rodnan skin score, units		1 (−0.4–3), 0.13	**9 (0.2–17), 0.045**	0.5 (−0.4–1), 0.27
Raynaud phenomenon		−5 (−47–38), 0.83	−23 (−226–181), 0.83	−2 (−23–18), 0.82
Digital ulcers		−4 (−40–33), 0.84	11 (−165–187), 0.90	0.01 (−18–18), 0.99
Calcinosis		−8 (−43–28), 0.67	−27 (−197–143), 0.75	−8 (−25–9), 0.36
Arthritis		33 (−10–77), 0.13	2 (−212–217), 0.98	−2 (−24–19), 0.83
Gastric reflux		−19 (−45–6), 0.14	−26 (−152–100), 0.69	−0.8 (−14–12), 0.90
Pathological esophageal manometry		−16 (−51–19), 0.35	−102 (−257–53), 0.19	−0.8 (−27–25), 0.95
Nailfold capillaroscopy pattern				
	Normal	ref.	ref.	ref.
	Pathological	18 (−22–57), 0.38	124 (−66–314), 0.20	−3 (−19–12), 0.68
Interstitial lung disease		14 (−20–48), 0.41	−7 (−174–160), 0.93	−5 (−22–12), 0.57
	FVC, %	−0.7 (−2–0.09), 0.081	−1 (−6–3), 0.53	0.1 (−0.3–0.5), 0.61
	FEV1, %	−0.5 (−2–0.6), 0.35	−0.8 (−6–5), 0.76	−0.1 (−0.6–0.3), 0.54
	DLCO, %	−0.5 (−1–0.5), 0.30	1 (−4–6, 0.67	−0.2 (−0.6–0.2), 0.39
Pulmonary hypertension		−12 (−51–27), 0.55	72 (−137–281), 0.49	9 (−8–25), 0.30
Anti-centromere antibody positivity		−17 (−46–12), 0.25	25 (−115–166), 0.72	−3 (−17–11), 0.65
Anti-Scl70 antibody		17 (−20–54), 0.36	−7 (−184–171), 094	3 (−15–21), 0.75
Therapies				
	Current NSAIDs	−11 (−50–29), 0.59	48 (−144–239), 0.62	**20 (0.9–39), 0.041**
	Current prednisone	24 (−10–58), 0.16	149 (−12–310), 0.070	13 (−3–30), 0.11
	Prednisone, mg/day	−6 (−28–16), 0.55	−31 (−139–77), 0.54	−0.5 (−9–8), 0.91
	Methotrexate	**59 (4–115), 0.037**	135 (−141–411), 0.33	4 (−24–32), 0.76
	Chloroquine	−18 (−76–39), 0.53	−100 (−377–177), 0.48	9 (−19–37), 0.53
	Bosentan	−17 (−83–49), 0.61	−6 (−325–313), 0.97	6 (−26–38), 0.71

In this analysis, sICAM-1 (soluble intercellular adhesion molecule-1), sVCAM-1 (soluble vascular cell adhesion molecule-1), and P-selectin are the dependent variables. The esophageal manometry assessment was available only for 33 patients. BMI: body mass index; SSc: systemic sclerosis; NSAIDs: non-steroidal anti-inflammatory drugs. FVC: forced vital capacity; FEV: forced expiratory volume; DLCO: diffusion capacity of the lung for the carbon monoxide. Significant *p*-values are depicted in bold.

**Table 3:** Cardiovascular-related data and their relation with sICAM-1, sVCAM-1, and P-selectin.

			sICAM-1, ng/mL			sVCAM-1, ng/mL			P-Selectin, ng/mL		
			Beta Coefficient (95% Confidence Interval)								
				*p*	*p*1		*p*	*p*2		*p*	*p*3
Metabolic syndrome, n (%)		47 (61)	19 (−7–45)	0.15	0.87	123 (−2–247)	0.054	0.21	8 (−5–21)	0.26	
Carotid atherosclerosis											
	Intima-media thickness, microns	663 ± 146	−0.02 (−0.1–0.07)	0.62		−0.3 (−0.7–0.1)	0.16	0.17	−0.008 (−0.05–0.04)	0.73	
	Plaque	28 (34)	26 (−4–57)	0.087	0.60	20 (−139–179)	0.80		2 (−13–17)	0.80	
SCORE2 calculator, %		4 (2–7)	1 (−2–4)	0.46		6 (−7–20)	0.36		0.3 (−1–2)	0.64	
SCORE2 categories, n (%)											
	Low to moderate	45 (56)	ref.	ref.		ref.	ref.		ref.	ref.	
	High	27 (33)	2 (−27–30)	0.90		7 (−130–145)	0.92		−7 (−21–7)	0.30	
	Very high	9 (11)	9 (−34–52)	0.69		94 (−114–302)	0.37		−2 (−23–19)	0.85	
Lipid profile											
CRP, mg/L		2.2 (0.8–4.6)	1 (−2–5)	0.50		−12 (−31–5)	0.16		0.7 (−1–3), 0.44	0.44	
CRP > 5, mg/L		34 (22)	−3 (−27–20)	0.79		−121 (−220–24)	0.23		−4 (−21–12)	0.63	
Cholesterol, mg/dL		207 ± 37	0.08 (−0.3–0.4)	0.66		−1 (−3–0.2)	0.088	0.10	−0.006 (−0.2–0.2)	0.95	
Triglycerides, mg/dL		187 ± 92	0.009 (−0.1–0.1)	0.90		−0.3 (1–0.4)	0.38		−0.006 (−0.08–0.06)	0.86	
HDL-cholesterol, mg/dL		52 ± 12	−0.5 (−2–0.5)	0.32		−3 (−8–2)	0.28		−0.2 (−0.7–0.3)	0.48	
LDL-cholesterol, mg/dL		118 ± 33	0.2 (−0.2–0.6)	0.43		−1 (−3–1)	0.32		0.03 (−0.2–0.2)	0.77	
LDL:HDL-cholesterol ratio		2.4 ± 0.9	8 (−8–24)	0.33		−21 (−100–57)	0.59		4 (−4–12)	0.35	
Non-HDL-cholesterol, mg/dL		155 ± 36	0.1 (−0.2–0.5)	0.43		−1 (−3–0.5)	0.16	0.17	0.02 (−0.2–0.2)	0.86	
Lipoprotein A, mg/dL		36 (13–91)	−0.04 (−0.2–0.1)	0.61		**−0.7 (−2–0.08)**	**0.077**	**0.020**	0.06 (−0.03–0.1)	0.17	0.15
Apolipoprotein A1, mg/dL		165 ± 27	−0.07 (−0.5–0.4)	0.77		−1 (−4–1)	0.27		−0.1 (−0.3–0.1)	0.34	
Apolipoprotein B, mg/dL		105 ± 25	0.2 (−0.3–0.7)	0.51		−2 (−5–0.5)	0.11	0.14	0.1 (−0.1–0.4)	0.38	
Apo B:apo A ratio		0.7 ± 0.2	20 (−44–85)	0.53		−153 (−468–162)	0.34		24 (−8–56)	0.14	0.16
Atherogenic index		4.2 ± 1.2	3 (−8–15)	0.55		−24 (−79–31)	0.39		2 (−4–8)	0.47	
Insulin resistance indices											
Glucose, mg/dL		98 ± 22	0.3 (−0.3–0.8)	0.38		−0.7 (−4–2)	0.62		0.1 (−0.2–0.4)	0.51	
Insulin, µU/mL		9.4 (4.9–18.0)	0.3 (−0.5–1)	0.43		**6 (3–10)**	**0.001**	**0.004**	0.3 (−0.1–0.7)	0.15	0.086
C-peptide, ng/mL		4.1 ± 3.2	3 (−0.8–7)	0.12	0.49	22 (3–41)	0.022	0.064	**2 (0.5–5)**	**0.013**	**0.010**
HOMA2-IR		1.3 (0.6–2.2)	2 (−4–9)	0.47		**45 (15–74)**	**0.003**	**0.012**	2 (−0.9–5)	0.16	0.086
HOMA2-S%		80 (45–160)	−0.4 (−0.1–0.04)	0.34		−0.2 (−0.6–0.2)	0.32		−0.02 (−0.06–0.03)	0.45	
HOMA2-B%-C-peptide		181 ± 122	0.02 (−0.08–0.1)	0.68		**0.7 (0.3–1)**	**0.003**	**0.007**	0.04 (−0.02–0.09)	0.18	0.17
Insulin resistance indices in nondiabetic patients (n = 74)											
HOMA2-IR		1.7 (1.0–11.2)	5 (−3–13)	0.20		71 (38–104)	**0.001**	**<0.001**	3 (−0.8–7)	0.12	0.091
HOMA2-S%		98 (47–174)	−0.05 (−0.1–0.04)	0.25		−0.03 (−0.7–0.1)	0.21		−0.02 (−0.06–0.03)	0.44	
HOMA2-B%-C-peptide		181 ± 126	0.03 (−0.08–0.1)	0.62		0.7 (0.3–1)	**0.002**	**0.005**	0.04 (−0.01–0.09)	0.13	0.16

Data represent the mean ± SD or the median (Q1–Q3) when the data are not normally distributed. In this analysis, sICAM (soluble intercellular adhesion molecule-1), vCAM-1 (vascular cell adhesion molecule-1), and P-selectin are the dependent variables. *p*1 is adjusted for sex, age, and body mass index, *p*2 is adjusted for age and body mass index, and *p*3 is adjusted for sex and current smoking. SCORE2: systematic coronary risk assessment; CRP: C-reactive protein; HDL: high-density lipoprotein; LDL: low-density lipoprotein. HOMA2-IR: insulin resistance index through homeostatic model assessment (calculated with glucose and insulin serum levels). HOMA2-S%: insulin sensitivity index through homeostatic model assessment (calculated with glucose and insulin serum levels). HOMA2-B%-C-peptide: β-cell function index through homeostatic model assessment (calculated with glucose and C-peptide serum levels). Significant *p* values are depicted in bold.

## Data Availability

Data will be made available upon request.

## Abbreviations

The following abbreviations are used in this manuscript:

SSc	Systemic sclerosis
cIMT	Carotid intima-media thickness
CAMs	Cell adhesion molecules
sICAM-1	Soluble intercellular adhesion molecule-1
sVCAM-1	Soluble vascular cell adhesion molecule-1
BMI	Body mass index
mRSS	Modified Rodnan skin score
FVC	Forced vital capacity
DLCO	Diffusing capacity of the lung for carbon monoxide
SCORE2	Cardiovascular risk score
HDL	High-density cholesterol
LDL	Low-density cholesterol
HOMA	Homeostatic model assessment
Q1–Q3	Interquartile range
NSAIDs	Non-steroidal anti-inflammatory drugs
FEV	Forced expiratory volume
CRP	C-reactive protein

## References

[1] Allanore Y., Simms R., Distler O., Trojanowska M., Pope J., Denton C.P., Varga J. (2015). Systemic Sclerosis. Nat. Rev. Dis. Primers.

[2] Arias-Nuñez M.C., Llorca J., Vazquez-Rodriguez T.R., Gomez-Acebo I., Miranda-Filloy J.A., Martin J., Gonzalez-Juanatey C., Gonzalez-Gay M.A. (2008). Systemic Sclerosis in Northwestern Spain: A 19-Year Epidemiologic Study. Medicine.

[3] Sobanski V., Giovannelli J., Allanore Y., Riemekasten G., Airò P., Vettori S., Cozzi F., Distler O., Matucci-Cerinic M., Denton C. (2019). Phenotypes Determined by Cluster Analysis and Their Survival in the Prospective European Scleroderma Trials and Research Cohort of Patients with Systemic Sclerosis. Arthritis Rheumatol..

[4] Ngian G.S., Sahhar J., Wicks I.P., Van Doornum S. (2011). Cardiovascular Disease in Systemic Sclerosis—An Emerging Association?. Arthritis Res. Ther..

[5] Au K., Singh M.K., Bodukam V., Bae S., Maranian P., Ogawa R., Spiegel B., McMahon M., Hahn B., Khanna D. (2011). Atherosclerosis in Systemic Sclerosis: A Systematic Review and Meta-Analysis. Arthritis Rheum..

[6] IMAJ | The Israel Medicine Association Journal | Volume 23, Number 4, April 2021 | Metabolic Syndrome in Systemic Sclerosis Patients: Data from Clinical Practice. https://www.ima.org.il/MedicineIMAJ/viewarticle.aspx?year=2021&month=04&page=262.

[7] Ferraz-Amaro I., Delgado-Frías E., Hernández-Hernández V., Sánchez-Pérez H., de Armas-Rillo L., Armas-González E., Machado J.D., Diaz-González F. (2021). HDL Cholesterol Efflux Capacity and Lipid Profile in Patients with Systemic Sclerosis. Arthritis Res. Ther..

[8] Park E.K., Lee S.G., Kim B.H., Park J.H., Lee S., Kim G.T. (2016). Insulin Resistance Is Associated with Digital Ulcer in Patients with Systemic Sclerosis. Clin. Exp. Rheumatol..

[9] Haydinger C.D., Ashander L.M., Tan A.C.R., Smith J.R. (2023). Intercellular Adhesion Molecule 1: More than a Leukocyte Adhesion Molecule. Biology.

[10] Kong D.H., Kim Y.K., Kim M.R., Jang J.H., Lee S. (2018). Emerging Roles of Vascular Cell Adhesion Molecule-1 (VCAM-1) in Immunological Disorders and Cancer. Int. J. Mol. Sci..

[11] McEver R.P. (2015). Selectins: Initiators of Leucocyte Adhesion and Signalling at the Vascular Wall. Cardiovasc. Res..

[12] Blankenberg S., Rupprecht H.J., Bickel C., Peetz D., Hafner G., Tiret L., Meyer J. (2001). Circulating Cell Adhesion Molecules and Death in Patients with Coronary Artery Disease. Circulation.

[13] Gross M.D., Bielinski S.J., Suarez-Lopez J.R., Reiner A.P., Bailey K., Thyagarajan B., Carr J.J., Duprez D.A., Jacobs D.R. (2012). Circulating Soluble Intercellular Adhesion Molecule 1 and Subclinical Atherosclerosis: The Coronary Artery Risk Development in Young Adults Study. Clin. Chem..

[14] Jenny N.S., Arnold A.M., Kuller L.H., Sharrett A.R., Fried L.P., Psaty B.M., Tracy R.P. (2006). Soluble Intracellular Adhesion Molecule-1 Is Associated with Cardiovascular Disease Risk and Mortality in Older Adults. J. Thromb. Haemost..

[15] Troncoso M.F., Ortiz-Quintero J., Garrido-Moreno V., Sanhueza-Olivares F., Guerrero-Moncayo A., Chiong M., Castro P.F., García L., Gabrielli L., Corbalán R. (2021). VCAM-1 as a Predictor Biomarker in Cardiovascular Disease. Biochim. Biophys. Acta Mol. Basis Dis..

[16] Sommer P., Schreinlechner M., Noflatscher M., Lener D., Mair F., Theurl M., Kirchmair R., Marschang P. (2023). Increasing Soluble P-Selectin Levels Predict Higher Peripheral Atherosclerotic Plaque Progression. J. Clin. Med..

[17] Bielinski S.J., Berardi C., Decker P.A., Kirsch P.S., Larson N.B., Pankow J.S., Sale M., de Andrade M., Sicotte H., Tang W. (2015). P-Selectin and Subclinical and Clinical Atherosclerosis: The Multi-Ethnic Study of Atherosclerosis (MESA). Atherosclerosis.

[18] Van Den Hoogen F., Khanna D., Fransen J., Johnson S.R., Baron M., Tyndall A., Matucci-Cerinic M., Naden R.P., Medsger T.A., Carreira P.E. (2013). 2013 Classification Criteria for Systemic Sclerosis: An American College of Rheumatology/European League against Rheumatism Collaborative Initiative. Ann. Rheum. Dis..

[19] Khanna D., Furst D.E., Clements P.J., Allanore Y., Baron M., Czirjak L., Distler O., Foeldvari I., Kuwana M., Matucci-Cerinic M. (2017). Standardization of the Modified Rodnan Skin Score for Use in Clinical Trials of Systemic Sclerosis. J. Scleroderma Relat. Disord..

[20] Smith V., Herrick A.L., Ingegnoli F., Damjanov N., De Angelis R., Denton C.P., Distler O., Espejo K., Foeldvari I., Frech T. (2020). Standardisation of Nailfold Capillaroscopy for the Assessment of Patients with Raynaud’s Phenomenon and Systemic Sclerosis. Autoimmun. Rev..

[21] Visseren F.L.J., MacH F., Smulders Y.M., Carballo D., Koskinas K.C., Bäck M., Benetos A., Biffi A., Boavida J.M., Capodanno D. (2021). 2021 ESC Guidelines on Cardiovascular Disease Prevention in Clinical Practice. Eur. Heart J..

[22] Touboul P.J., Hennerici M.G., Meairs S., Adams H., Amarenco P., Bornstein N., Csiba L., Desvarieux M., Ebrahim S., Hernandez Hernandez R. (2012). Mannheim Carotid Intima-Media Thickness and Plaque Consensus (2004-2006-2011). An Update on Behalf of the Advisory Board of the 3rd, 4th and 5th Watching the Risk Symposia, at the 13th, 15th and 20th European Stroke Conferences, Mannheim, Germany, 2004, Brussels, Belgium, 2006, and Hamburg, Germany, 2011. Cerebrovasc. Dis..

[23] Wallace T.M., Levy J.C., Matthews D.R. (2004). Use and Abuse of HOMA Modeling. Diabetes Care.

[24] Lee S.-G., Kim J.-M., Lee S.-H., Kim K.-H., Choi S.-A., Park E.-K., Jung W.-J., Park Y.-E., Park S.-H., Lee J.-W. (2012). Frequency of Metabolic Syndrome in Female Patients with Systemic Sclerosis: A Preliminary Report. J. Rheum. Dis..

[25] Mangoni A.A., Zinellu A. (2024). Circulating Cell Adhesion Molecules in Systemic Sclerosis: A Systematic Review and Meta-Analysis. Front. Immunol..

[26] Veale D.J., Kirk G., McLaren M., Belch J.J.F. (1998). Clinical Implications of Soluble Intercellular Adhesion Molecule-1 Levels in Systemic Sclerosis. Rheumatology.

[27] Stratton R.J., Coghlan J.G., Pearson J.D., Burns A., Sweny P., Abraham D.J., Black C.M. (1998). Different Patterns of Endothelial Cell Activation in Renal and Pulmonary Vascular Disease in Scleroderma. QJM.

[28] Denton C.P., Bickerstaff M.C.M., Shiwen X., Carulli M.T., Haskard D.O., Dubois R.M., Black C.M. (1995). Serial Circulating Adhesion Molecule Levels Reflect Disease Severity in Systemic Sclerosis. Br. J. Rheumatol..

[29] Hsu L.A., Ko Y.L., Wu S., Teng M.S., Chou H.H., Chang C.J., Chang P.Y. (2009). Association of Soluble Intercellular Adhesion Molecule-1 with Insulin Resistance and Metabolic Syndrome in Taiwanese. Metabolism.

[30] Tso T.K., Huang W.-N. (2007). Elevated Soluble Intercellular Adhesion Molecule-1 Levels in Patients with Systemic Lupus Erythematosus: Relation to Insulin Resistance. J. Rheumatol..

[31] Matsumoto K., Sera Y., Abe Y., Ueki Y., Miyake S. (2001). Serum Concentrations of Soluble Vascular Cell Adhesion Molecule-1 and E-Selectin Are Elevated in Insulin-Resistant Patients With Type 2 Diabetes. Diabetes Care.

[32] Yang J.-W., Day Y.-J., Hung L.-M. (2014). P-Selectin Deficiency Protects against High-Fat Diet-Induced Obesity and Insulin Resistance (641.1). FASEB J..

[33] Chen N.-G., Holmes M., Reaven G.M. (1999). Relationship Between Insulin Resistance, Soluble Adhesion Molecules, and Mononuclear Cell Binding in Healthy Volunteers. J. Clin. Endocrinol. Metab..

